# Comparative *De Novo* Transcriptome Analysis of Fertilized Ovules in *Xanthoceras sorbifolium* Uncovered a Pool of Genes Expressed Specifically or Preferentially in the Selfed Ovule That Are Potentially Involved in Late-Acting Self-Incompatibility

**DOI:** 10.1371/journal.pone.0140507

**Published:** 2015-10-20

**Authors:** Qingyuan Zhou, Yuanrun Zheng

**Affiliations:** Key Laboratory of Plant Resources, Institute of Botany, Chinese Academy of Sciences, Beijing, China; Wuhan University, CHINA

## Abstract

*Xanthoceras sorbifolium*, a tree species endemic to northern China, has high oil content in its seeds and is recognized as an important biodiesel crop. The plant is characterized by late-acting self-incompatibility (LSI). LSI was found to occur in many angiosperm species and plays an important role in reducing inbreeding and its harmful effects, as do gametophytic self-incompatibility (GSI) and sporophytic self-incompatibility (SSI). Molecular mechanisms of conventional GSI and SSI have been well characterized in several families, but no effort has been made to identify the genes involved in the LSI process. The present studies indicated that there were no significant differences in structural and histological features between the self- and cross-pollinated ovules during the early stages of ovule development until 5 days after pollination (DAP). This suggests that 5 DAP is likely to be a turning point for the development of the selfed ovules. Comparative *de novo* transcriptome analysis of the selfed and crossed ovules at 5 DAP identified 274 genes expressed specifically or preferentially in the selfed ovules. These genes contained a significant proportion of genes predicted to function in the biosynthesis of secondary metabolites, consistent with our histological observations in the fertilized ovules. The genes encoding signal transduction-related components, such as protein kinases and protein phosphatases, are overrepresented in the selfed ovules. *X*. *sorbifolium* selfed ovules also specifically or preferentially express many unique transcription factor (TF) genes that could potentially be involved in the novel mechanisms of LSI. We also identified 42 genes significantly up-regulated in the crossed ovules compared to the selfed ovules. The expression of all 16 genes selected from the RNA-seq data was validated using PCR in the selfed and crossed ovules. This study represents the first genome-wide identification of genes expressed in the fertilized ovules of an LSI species. The availability of a pool of specifically or preferentially expressed genes from selfed ovules for *X*. *sorbifolium* will be a valuable resource for future genetic analyses of candidate genes involved in the LSI response.

## Introduction

Self-incompatibility (SI) is considered to be the most important and widespread mechanism promoting outcrossing in flowering plants. Various SI systems have been described in which self-fertilization can be prevented at any stage from the first contact of the pollen and the stigma to the fertilization of the ovule, indicating considerable diversity of SI mechanisms within plants [1].

The genetically characterized SI systems fall into two broad categories: gametophytic and sporophytic self-incompatibility (GSI and SSI) [2]. All characterized SSI systems show inhibition of incompatible pollen on the stigma surface, whereas in GSI systems, inhibition of incompatible pollen tubes frequently occurs within the style. In addition to these two SI systems, there are ovarian or late-acting SI systems (OSI or LSI), in which self-pollinated flowers consistently fail to form fruits or seeds, despite the fact that pollen tubes grew to the ovaries and penetrated the ovules; however, the ovules are rejected either just before fertilization or at some stage after fertilization [3–4].

A major goal of recent research into SI has been to identify and characterize genes that control SI. The molecular mechanisms of GSI and SSI reactions have been established in detail in only five families, but these families are widely diverse [5]. Most of the genetically well-characterized GSI and SSI systems are controlled by a single locus with multiple alleles, the *S* locus. The *S*-locus comprises at least two tightly linked, polymorphic genes, one of which encodes the male determinant and the other encodes the female determinant. In the GSI families Solanaceae, Rosaceae and Plantaginaceae, stylar inhibition of an incompatible pollen tube is mediated through an interaction between a stylar S-RNase (female determinant) and a pollen tube-borne F-box protein (male determinant), SLF or SFB, in which incompatibility degrades pollen tube RNA [6–8]. In addition to these primary male and female determinants, many other genes are also known or predicted to reside at the *S* locus [9]. Genetic data also show that other pistil factors not linked to the *S* locus, such as a small asparagine-rich protein (HT-B), 120K, and 4936 factor, are important for fully functional SI [10].

In GSI in the Papaveraceae family, the SI female determinant is a stigma-expressed S-glycoprotein ligand, a small extracellular signaling molecule named PrsS. The male determinant is a pollen-expressed Ca^2+^-channel protein named PrpS. With incompatible pollinations, PrsS reacts with its cognate trans-membrane receptor PrpS to trigger a Ca^2+^ -dependent signaling cascade, resulting in the inhibition of pollen tube growth [11–12].

In SSI in *Brassica*, the principal female determinant is a stigma-specific S-locus receptor kinase (SRK) that consists of an extracellular domain in the stigmatic pellicle, a trans-membrane domain and an intracellular serine/threonine kinase domain [13–14]. The male determinant is a small cysteine-rich protein (<10 kDa, termed SP11 or SCR) located in the pollen coat [15–16]. SSI is regulated by an S-haplotype-specific protein interaction in which SRK is activated by its cognate ligand SCR/Sp11 leading to an intracellular signal transduction cascade [17]. This interaction involves the formation of a receptor complex involving SRK, SCR and a cytoplasmic kinase, MLPK (M-locus protein kinase), and autophosphorylation of SRK [18–19].

Compared to GSI and SSI, the genetic mechanisms of LSI are poorly understood because genetic analysis of late-acting SI is more difficult than that of prezygotic SI [4]. In addition, the presence of LSI has often been correlated with a woody perennial habit [20]. The perennial nature of trees with LSI makes genetic analysis very time-consuming or impracticable [4,21].

Despite the difficulties inherent in the genetic characterization of LSI, a limited number of studies showed that LSI is under genetic control [22–23]. The genetic basis of LSI systems is frequently hypothesized to be gametophytic [24]. Crossing experiments in several species suggested that the LSI response is controlled by at least one locus and is most likely controlled by multiple loci with multiple alleles [2,23,25–26]. However, no effort has been made to identify the genes of the SI loci, so the genes for the loci on both the pollen and pistil sides remain unknown.

*Xanthoceras sorbifolium*, a tree species of Sapindaceae endemic to northern China, is an oilseed crop that has high oil content of up to 40%. Its seed oil is of good quality for dietary applications because of its high unsaturated fatty acid content. In addition, it fulfills many of requirements for biodiesel production and is recognized as an important biodiesel crop in China. *X*. *sorbifolium* is characterized by late-acting self-incompatibility [27]. Pollen tubes penetrate ovules and effect double fertilization after self-pollination, but selfed ovules are uniformly rejected at the endosperm syncytial stage [27].

Little information is available regarding the molecular mechanisms regulating the LSI response. The aims of the present study were to identify candidate genes involved in pollen-ovule interactions and LSI mechanisms. These genes have been identified by comparing the transcriptomes of the self- and cross-pollinated ovules of *X*. *sorbifolium* at the whole genome level using high-throughput next-generation sequencing technology to perform an RNA-seq analysis. To achieve this goal and to gain a better understanding of the LSI process in *X*. *sorbifolium*, further detailed investigations of the histology of developing ovules and observations of LSI phenomena were also performed.

## Materials and Methods

### Plant materials

Three cultivated ten-year-old *X*. *sorbifolium* trees were used in this study. All the experimental materials, including young ovules at various stages of development and fertilized ovules after self- and cross-pollination, were harvested from the three unrelated trees A, B, and C.

### Morphological and histological analysis

The young, fertilized ovules were dissected from pistils and fixed in formalin—acetic acid—alcohol (FAA) for light and scanning electron microscopy (LM and SEM), respectively. Fixed specimens for LM were dehydrated through a tertiary butyl alcohol series, embedded in paraffin, and sectioned at 6–8 μm. Sections were stained with 1.0% aqueous safranin and 0.05% fast green. For SEM, fixed specimens were dehydrated in an alcohol series, critical point dried, coated with gold, and viewed with a Hitachi S-4800 scanning electron microscope.

### RNA extraction, library construction and Illumina sequencing

For the RNA-seq sampling, ovules were harvested from the pistils that were self- and cross-pollinated at 5 DAP. Each pollination treatment was represented by three biological replicates from the trees A, B and C, resulting in a total of four samples from two pollination treatments. Each replicate consisted of 6 ovules taken from random positions on each of ten bunches on that tree. The samples were flash frozen in liquid nitrogen and stored at −80°C until further use.

The total RNA from the ovules with the two pollination treatments was extracted using Trizol Reagent (Invitrogen, Carlsbad, CA, USA) and purified using an RNeasy Mini Kit (Qiagen, Hilden, Germany) according to the manufacturers’ protocols. The quality of the total RNA was determined using a NanoDrop 2000 Spectrophotometer (Thermo Fisher, USA). The mRNA was purified from the total RNA samples using a Dynabead mRNA Purification Kit according to the manufacturer’s instructions (Invitrogen, Carlsbad, CA, USA), and the quality was assessed using an Agilent 2100 Bioanalyzer (Agilent Technologies, Inc., Waldbronn, Germany). Double-stranded cDNA was synthesized using the SuperScript Double-Stranded cDNA Synthesis Kit (Invitrogen, Carlsbad, CA, USA). Specific adapters were ligated to the fragmented cDNA and denatured to generate single-stranded cDNA followed by emulsion PCR amplification. The sequencing was performed using an Illumina HiSeq 2000 sequence analyzer at Hanyu Genomics Institute (Shanghai, China). RNA-seq read data were deposited in the NCBI Sequence Read Archive (SRA) under accession number SRP062402.

### *De novo* transcriptome assembly

The raw reads were filtered to obtain high quality *de novo* transcriptome sequence data. We discarded the reads with adapter contamination, those with more than 5% unknown nucleotides, and those of low quality (≥20% of the bases with a quality score (Q)≤10) using a Perl script. Clean reads were assembled using the Trinity *de novo* assembler (http://trinityrnaseq.sourceforge.net/) [28]. Trinity is a 3-module assembler composed of inchworm, chrysalis and butterfly. Inchworm assembles clean reads into a set of full length linear contigs for the major isoform as well as unique portions of minor spliced variants. Chrysalis then connects these isoforms into components that were likely to represent alternative splice forms and closely related paralogs by finding shared subsequences in the contigs, and build de Bruijn transcript graphs for each component. Finally, butterfly processes each generated graph and enumerates full length alternatively spliced isoforms and transcripts from paralogous genes. The following parameters were used in Trinity: min_glue = 3, V = 10, edge-thr = 0.05, min_kmer_cov = 3, path_reinforcement_distance = 85, group_pairs_distance = 250, and the other parameters were set as the default.

The assembled contigs that were shorter than 200 bp were removed using the Perl script seqclean. Redundant contigs were trimmed by using a self-cross BlastN, searching with a cutoff E value ≤1×10^−10^, identity ≥95%, covered length ≥90%. Compared with ribosomal RNA (rRNA) databases (http://www.girinst.org), the contigs meeting a cutoff (E value ≤1×10^−10^, identity ≥80% and covered length of query ≥80%) were removed. We identified and discarded any potential contaminated sequence from microbes using BlastN against databases of microbial genomes downloaded from NCBI (ftp://ftp.ncbi.nih.gov/refseq/release/microbial/) with the cutoff E value ≤1×10^−10^, identity ≥80%, and alignment length ≥90%.

### Functional annotation and classification

All assembled unigenes were annotated with getorf from the EMBOSS package. The ORFs were then aligned with Swiss-Prot and NCBI NR peptide databases with thresholds of E-value = 1e-5 and the ORF with the highest score was used to annotate the contig. For the contigs that did not have hits in these databases, the longest ORF were annotated as ‘‘hypothetical protein”. Domain-based alignments were performed against the KOG database at NCBI with a cutoff E-value = 1e-5. GO annotations were carried out using the Blast2GO software. WEGO software (http://wego.genomics.org.cn/cgi-bin/wego/index.pl) was used to produce GO functional classification for all unigenes [29]. Another GO analysis tool, SEA, was used to identify overrepresented GO terms [30]. KEGG pathways annotations were performed using the KEGG Automatic Annotation Server (KAAS) (http://www.genome.jp/kaas-bin/kaas_main?modeest_b) with the bi-directional best hit information method [31]. KAAS annotates every submitted sequence with KEGG orthology (KO) identifiers that represent an orthologous group of genes directly linked to an object in the KEGG pathways and BRITE functional hierarchy [31–32]. Transcription factors (TFs) were analyzed with all the unigenes by BLASTX searches against the Plant Transcription Factor Database (PlnTFDB) (version 3.0) (E-value = 1e-10).

### Analysis of differentially expressed genes (DEGs)

To identify differentially expressed genes in the selfed and crossed ovules, a rigorous algorithm was developed based on the method described by Audic and Claverie [33]. The number of reads for each of the contigs from the samples of two pollination treatments was converted to reads per kilobase per million (RPKM). The false discovery rate was used to determine the threshold of the P-value in multiple tests and analyses. We used an FDR of < 1e-05, the absolute value of log2 ratio > 2, and fold change >1 as thresholds to define significant differences in gene expression [34].

### Quantitative real time reverse transcription-PCR analysis

Quantitative RT-PCR was carried out on cDNA generated from three biological replicates harvested as described above, one of which corresponded to the sample subjected to Illumina sequencing for RNA-seq analysis. The total RNA (10 μg) was reverse-transcribed with an oligo (dT) primer for cDNA synthesis using a SuperScript III First-Strand Synthesis Kit (Invitrogen). Amplification of the *X*. *sorbifolium* actin gene was used as an internal control to normalize all data. Gene-specific primers were designed using PRIMEREXPRESS software (Applied Biosystems). The primer sequences are listed in S3 Table. Quantitative PCR assays were performed in three technical replications using SYBR Green Real-time PCR Master Mix (Toyobo, Osaka, Japan) with a Bio-Rad CFX96 Real-Time Detection System. The quantitative variation in the different replicates was calculated using the delta delta threshold cycle relative quantification method.

## Results

### Structural and histological analyses of ovule development

**Fig 1:**
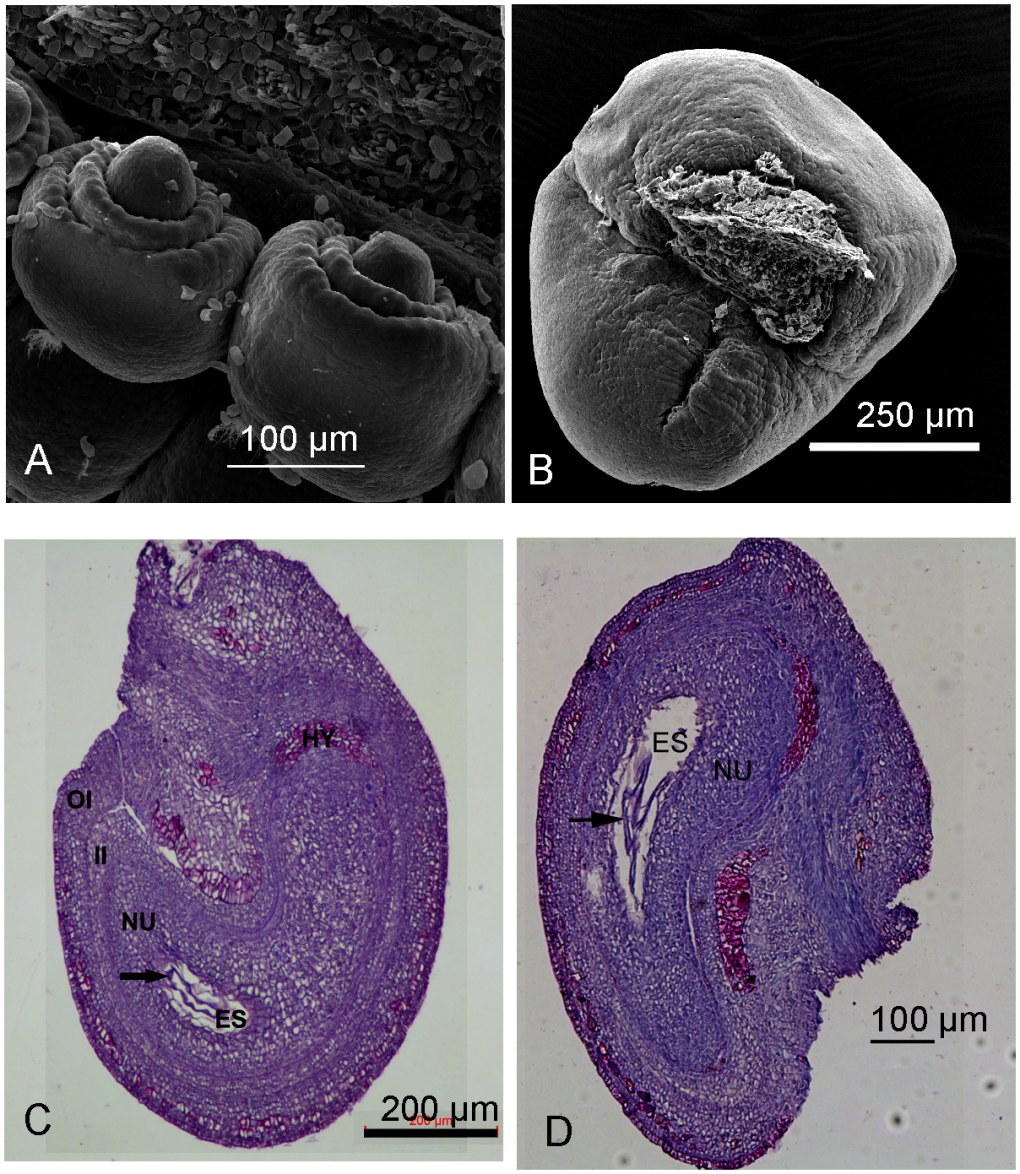
Developing ovules of *Xanthoceras sorbifolium*. A, B. Scanning electron microscope images. A: Young ovules from a floral bud. B: A selfed ovule at 5 d after pollination (DAP). C and D: Longitudinal section of crossed and selfed ovules at 5 DAP, respectively. Arrows show free nuclear endosperm. Abbreviations: ES, embryo sac; HY, hypostase; NU, nucellus; OI, outer integument; II, inner integument.

The mature ovules of *X*. *sorbifolium* consisted of a short funicle, an inner and outer integument, a nucellus and an embryo sac (Fig 1A, 1B, 1C and 1D). There were 9 to 12 layers of cells in the outer integument and 4 to 7 layers in the inner integument at 5 DAP (Fig 1C and 1D). The innermost 3 to 5 layers of the outer integument on the funicular side gave rise to many lignified cells that contained tannin-like substances after fertilization. Later, several layers of cells on the abaxial side also produced lignified cell walls and accumulated tannin-like substances. The integumentary parenchyma became compressed and was absorbed following expansion of the fertilized embryo sac.

The nucellus was well developed and exhibited conspicuous curvature in the fertilized ovules. A beak-shaped outgrowth pointing toward the micropyle (nucellus beak) was formed at the nucellar apex. A massive, cup-like hypostase situated above the chalazal vasculature was differentiated at the nucellar and chalazal tissue (Fig 1C). The hypostase cells became filled with tannin-like substances after fertilization, with the cell walls thickening with suberin and lignin deposition.

A large embryo sac was embedded within the massive nucellar tissue. After fertilization, the embryo sac expanded dramatically in both length and width and became progressively curved. The primary endosperm nucleus migrated to the basal cytoplasm of the central cell and divided without formation of interzone phragmoplasts or a cell wall between sister nuclei within 30 h after self- and cross-pollination. Mitosis continued more or less synchronously and approximately two hundred free nuclei were generated by 5 DAP. The nuclei in the endosperm coenocyte became evenly dispersed in a thin layer of cytoplasm around the periphery of a large central vacuole. Resting zygotes were observed at 5 DAP, and they never divided in the selfed ovules.

### Morphological observations of late acting self-incompatibility

**Fig 2:**
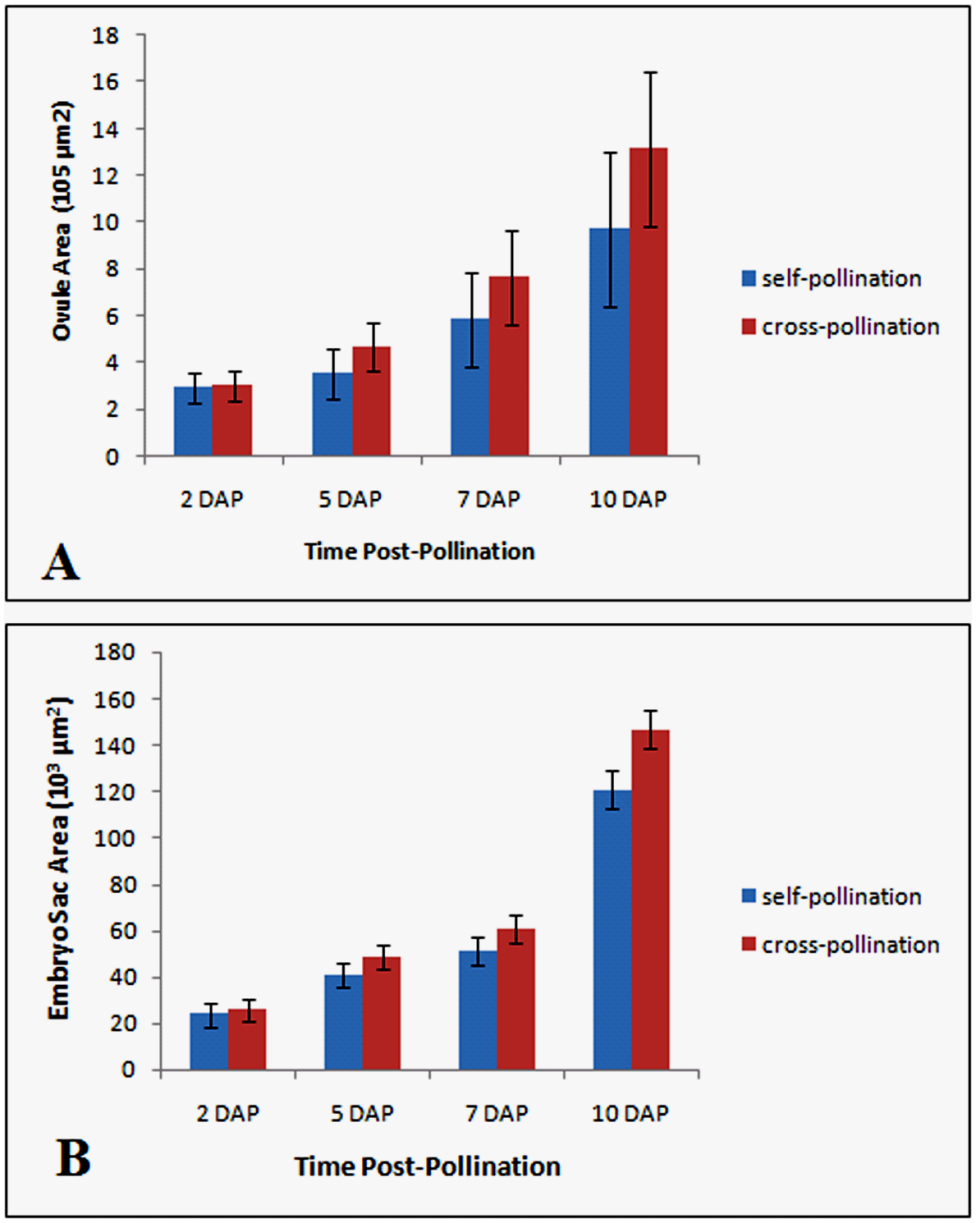
Mean area of the median longitudinal section of ovule and embryo sac after cross- and self- pollination of *Xanthoceras sorbifolium*. A: Ovule area; B: Embryo sac area.

All the ovules in an ovary were penetrated by pollen tubes, followed by double fertilization after either self- or cross- pollination. Ovule development within an ovary was homogeneous during the first 5 days after pollination. However, some of the ovules ceased developing and showed visible signs of degeneration at 6 d after pollination. The mean area of the median longitudinal section of the ovules and embryo sacs was significantly smaller after self-pollination than it was after cross-pollination at 7 DAP (Fig 2A and 2B). Lignification of the cell wall and accumulation of tannin-like substances in the outer integument also occurred more quickly and more heavily after self- than cross-pollination. A greater proportion of ovules degenerated after self- than cross-pollination by 8 DAP. All of the ovules in an ovary degenerated 8–15 d after self-pollination depending on the tree. The pistils in which more than 60% of the ovules degenerated would abort after either self- or cross-pollination.

### Identification of genes expressed specifically or preferentially in the selfed ovules

**Fig 3:**
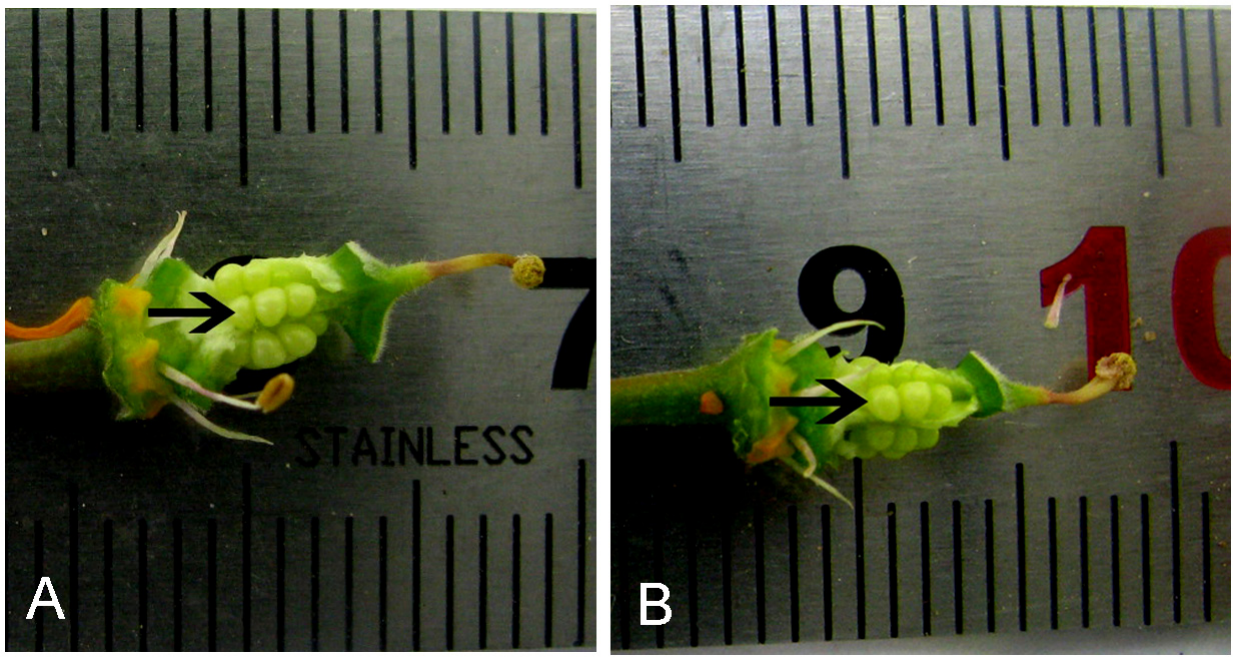
The selfed and crossed ovules were harvested from the pistil at 5 d after pollination for the study of RNA-seq and morphology. A and B showing the crossed and selfed ovules (arrows), respectively.

To identify the genes involved in selfed ovule rejection and late acting self-incompatibility, we performed a comparative RNA-seq analysis on the selfed and crossed ovules of *X*. *sorbifolium*. These two samples enabled us to distinguish selfed ovule specific or preferential transcripts from transcripts that contribute to common biological processes and cellular activities. As stated above, there are no discernable differences in structural and histological features between the selfed and crossed ovules during the early stage of development until 5 DAP. After that time, the selfed ovules begin to show differences in histology compared with the crossed ones. These observations suggest that 5 DAP is likely to be a turning point for development of selfed ovules and that the reprogrammed gene expression at this stage possibly represents the molecular mechanisms modulated by the LSI genes that may play crucial roles in ovule development. Thus, selfed and crossed ovules at 5 DAP were sampled for the present study of comparative transcriptomics (Fig 3A and 3B).

**Fig 4:**
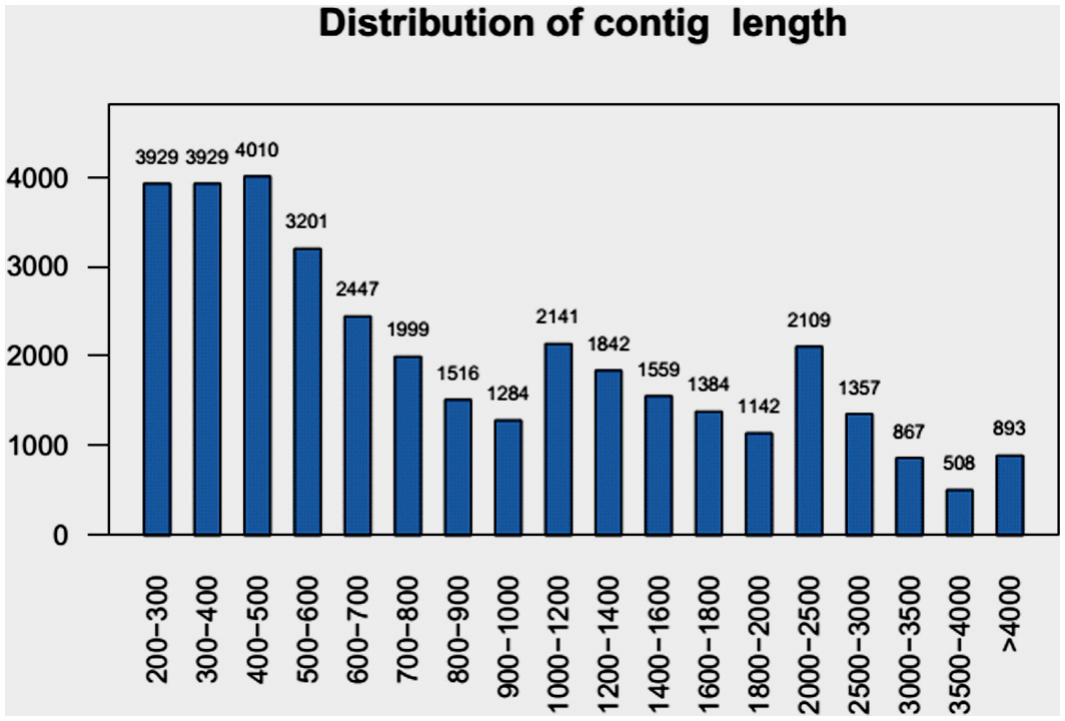
Distribution of contig length. The X-axis indicates contig length (bp). The Y-axis indicates number of unigenes.

The mRNA from the samples of two pollination treatments was used to construct cDNA libraries, which were then sequenced on an Illumina HiSeq 2000 system. A total of 35.8 and 24.1 million raw reads were generated from the selfed and crossed ovules, respectively. After removing low quality reads, including reads with adapter sequences, those with unknown nucleotides comprising more than 5%, and those of low quality (≥20% of the bases with a quality score (Q)≤10), 34,743,319 and 23,135,380 high-quality reads were obtained from the selfed and crossed ovules, respectively. All of the high-quality reads were pooled together for *de novo* transcriptome assembly into contigs using Trinity software (version v2013-02-25). The assembled sequences were then filtered to remove the contigs that were shorter than 200 bp, those that were either viral or bacterial in origin, those that contained redundant sequences, and those that contained ribosomal RNA sequences. Ultimately, we obtained 36,117 unigenes that ranged in length from 201 to 32,445 bp. The average and N50 lengths of the unigenes were 1,137 and 1,771 bp, respectively. The length distribution of unigenes is shown in Fig 4. Approximately forty-six percent of the unigenes (16,602) were longer than 800 bp.

Using both a fold change of >1 and a false discovery rate (FDR) of <1e-05 as a cutoff to identify differentially expressed genes, 274 unigenes were found that were significantly up-regulated in the selfed ovules compared to the crossed ovules (S1 Table). These up-regulated unigenes were considered to be specifically (fold change >5) or preferentially expressed in the selfed ovules (hereafter designated as the selfed ovule dataset). Using the same standard, we also identified 42 unigenes specifically or preferentially expressed in the crossed ovules (S2 Table).

### Confirmation of genes expressed specifically or preferentially in the selfed ovule by quantitative real-time RT-PCR analysis

**Fig 5:**
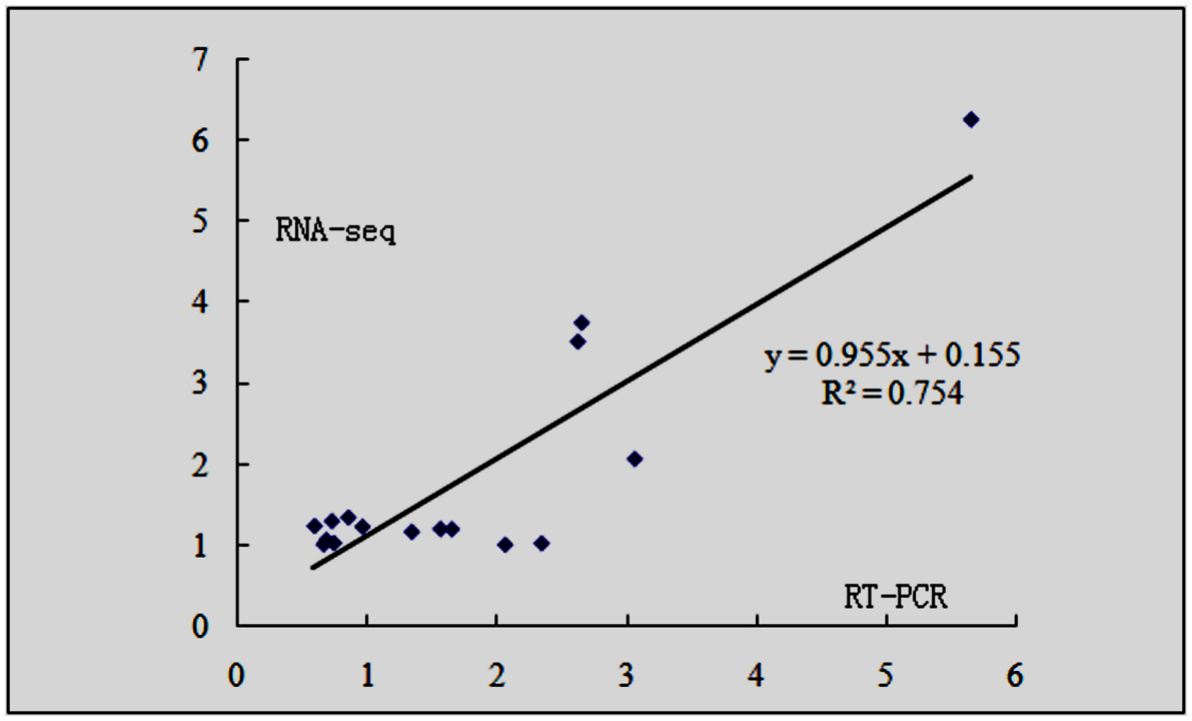
Validation of RNA-seq results by quantitative real time RT-PCR. Correlation plots indicating the relationship between qPCR results (fold change; Y-axis) of 16 selected genes expressed in the selfed and crossed ovules and the corresponding data from RNA-seq analysis (X-axis).

To validate the RNA-seq data and to check whether the candidate genes are specifically or preferentially expressed in the selfed ovules, 16 genes were randomly selected from the selfed ovule dataset for quantitative real-time RT-PCR analysis (the primer sequences are available in S3 Table). The expression of all tested genes was validated using PCR in the selfed and crossed ovules. Scatterplots were generated by comparing the log2-fold change determined by the transcriptome analysis and quantitative real-time RT-PCR. The correlation between these two analyses was then evaluated. The results showed that the expression patterns of these genes examined using quantitative real-time RT-PCR were well correlated with those by RNA-seq (R^2^ = 0.754), thus verifying the reliability of the RNA-seq technique (Fig 5).

### Functional annotation of the transcriptome gene models

The ORFs of all the gene models were predicted using getorf (EMBOSS 6.2.0). A total of 36,101 (99.96%) unigenes were predicted to have ORFs longer than 30 amino acids (aa). The ORF of each predicted protein was aligned against the Swiss-Prot and NCBI non-redundant (NR) databases using BLASTP with an E-value cutoff of 1e-5. The homology search results showed that 16,714 (46.27%) and 9,106 (25.21%) of the 36,117 *X*. *sorbifolium* unigenes had significant matches with sequences in the NCBI NR and Swiss-Prot protein databases, respectively. Altogether, 16,722 (46.30%) unigenes were successfully annotated using these two public databases. The species distribution of the best match for each NR annotated unigene showed 5,604 (33.53%) matches with *Vitis vinifera* sequences, 4,351 (26.03%) with *Ricinus communis*, 3,928 (23.50%) with *Populus trichocarpa*, 818 (4.89%) with *Glycine max*, 284 (1.70%) with *Arabidopsis*, and 202 (1.21%) with *Medicago*.

**Fig 6:**
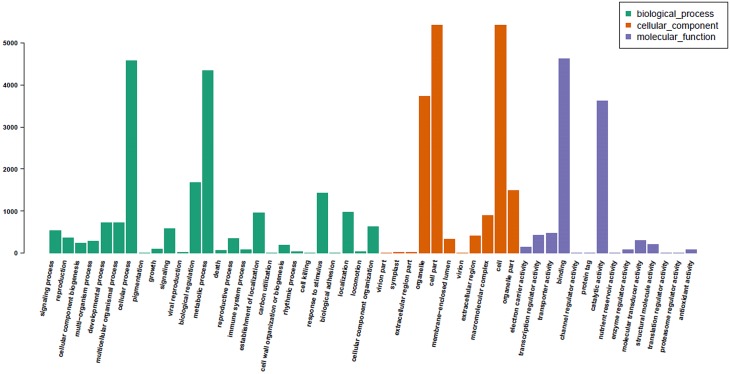
Gene Ontology categories of assembled unigenes. Unigenes were assigned to three main categories (biological processes, cellular components, and molecular functions) and 51 subcategories. The X-axis indicates GO term. The Y-axis indicates number of unigenes.

The assembled unigenes were further annotated with Gene Ontology (GO) terms. Of the 36,117 unigenes, 6,809 sequences were assigned one or more GO terms (S4 Table). These 6,809 unigenes were categorized into 51 GO functional groups under three main categories: molecular function, biological process and cellular component (Fig 6). Within the biological process category, the terms cellular process, metabolic process, and biological regulation were dominant. In the cellular component category, most unigenes were assigned to cell, cell part, and organelle. In the molecular function category, the most highly represented GO terms were binding and catalytic activity.

**Fig 7:**
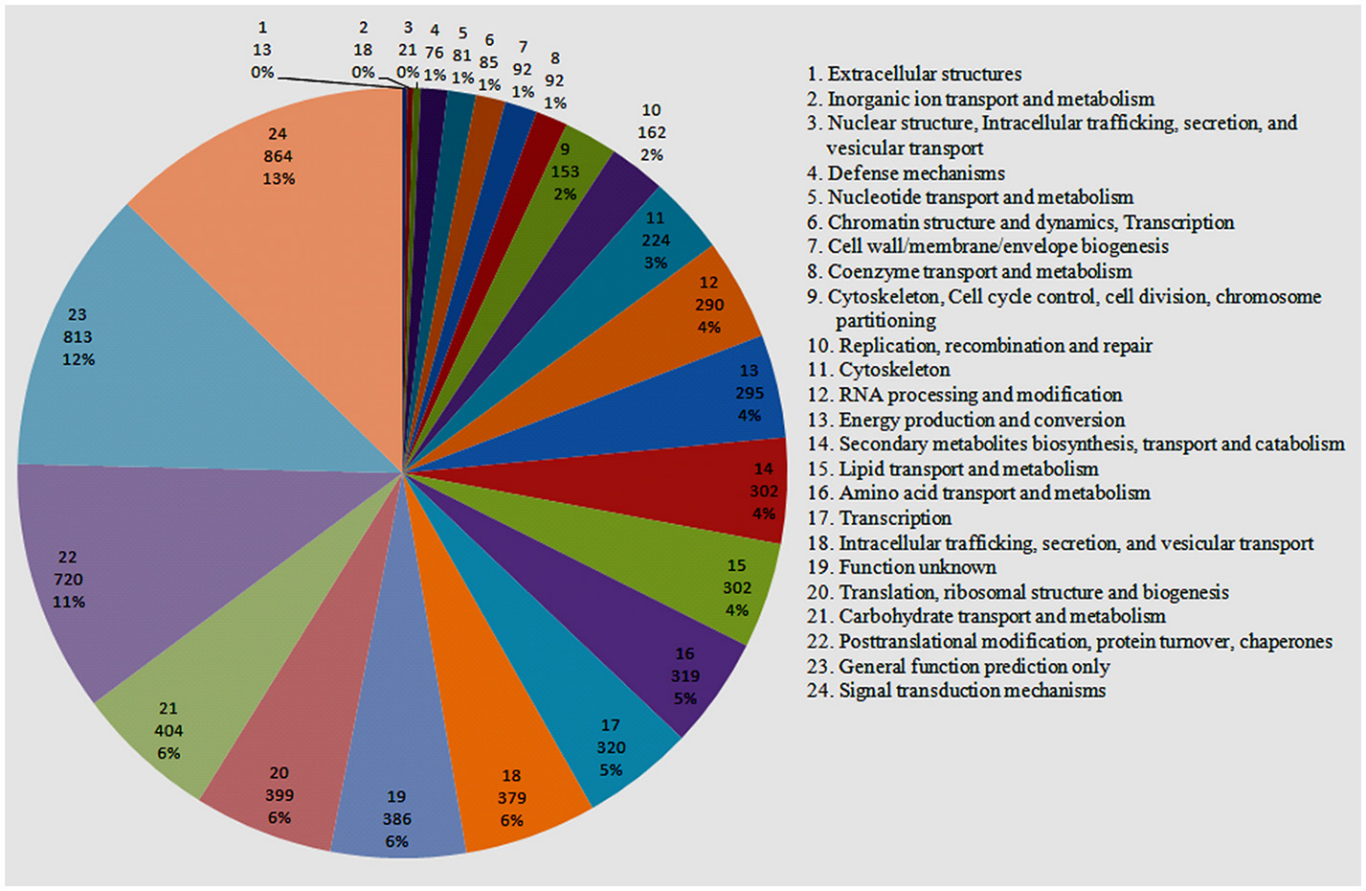
KOG function classification. The unigenes were aligned to the KOG database to predict and categorize possible functions. A total of 6343 unigenes were assigned to 24 categories.

All unigenes were further annotated and classified based on EuKaryotic Orthologous Groups (KOG) category. A total of 6,343 unigenes were assigned KOG functional annotation and grouped into 24 functional categories (Fig 7). Among these categories, signal transduction mechanisms (864, 13.62%); general function prediction only (813, 12.81%); and posttranslational modification, protein turnover, chaperones (720, 11.35%) were dominant, followed by carbohydrate transport and metabolism (404, 6.37%), and translation, ribosomal structure and biogenesis (399, 6.29%). For the category signal transduction mechanisms, the most abundant type of unigene was serine/threonine protein kinases (310, 35.92%).

We also annotated 5,696 unigenes with a K number to BRITE functional hierarchies, and 3,724 of them were assigned with an EC number (S5 Table). The BRITE functional mapping revealed the most common classifications and categorized the unigenes into 252 KEGG pathways (S6 Table). Among these pathways, ribosome (164), biosynthesis of amino acids (158), carbon metabolism (134), protein processing in endoplasmic reticulum (124), spliceosome (117), RNA transport (108), phagosome (100), purine metabolism (97), RNA polymerase (97), and plant hormone signal transduction (91) were most highly represented.

### Functional annotation of genes expressed differentially in the selfed- and crossed ovules

**Fig 8:**
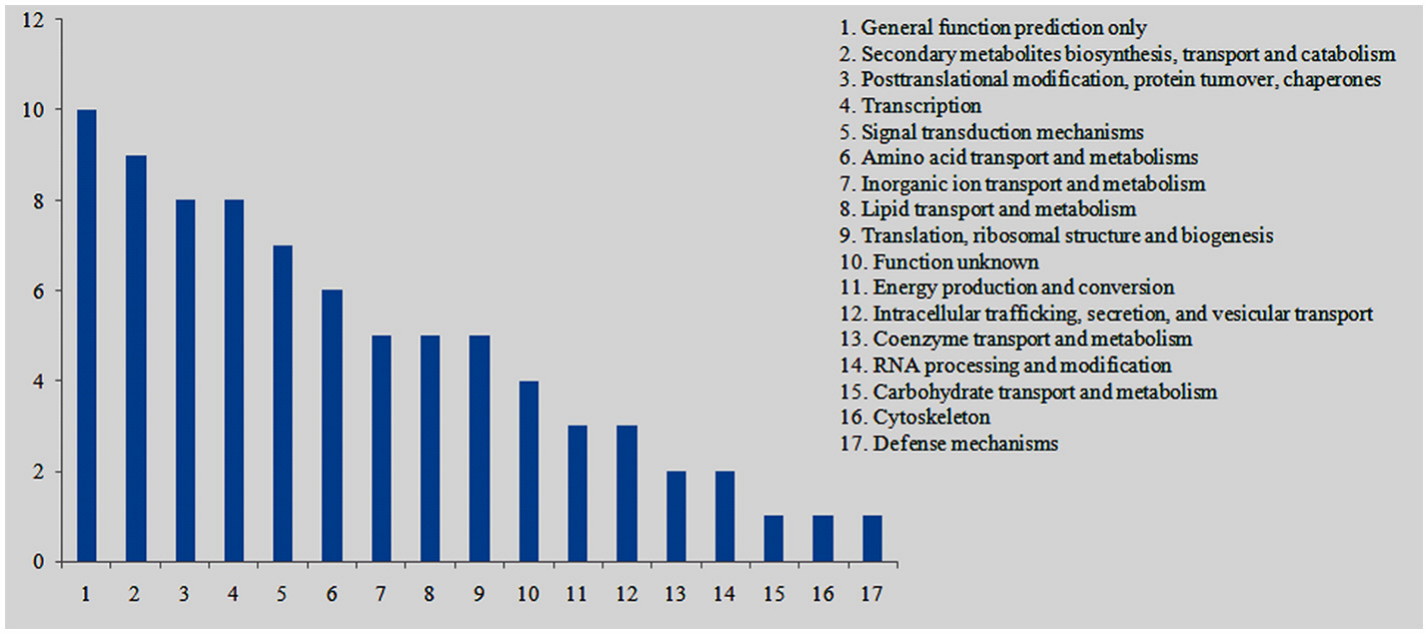
KOG function classification of the genes expressed specifically or preferentially in the selfed ovules of *Xanthoceras sorbifolium*. The X-axis indicates KOG function classification. The Y-axis indicates number of unigenes.

Of the 274 genes that were specifically or preferentially expressed in the selfed ovules, 76 were annotated with KOG functions (S7 Table). The 76 genes with KOG annotation were grouped into 17 functional categories. The five largest categories were general function prediction only (13.16%); secondary metabolites biosynthesis, transport and catabolism (11.84%); posttranslational modification, protein turnover, chaperones (10.53%); transcription (10.53%); and signal transduction mechanisms (9.21%) (Fig 8). The most abundant genes in the second largest category encode cytochrome P450 monooxygenases, of which 3 belong to the cytochrome P450 CYP2 family.

**Fig 9:**
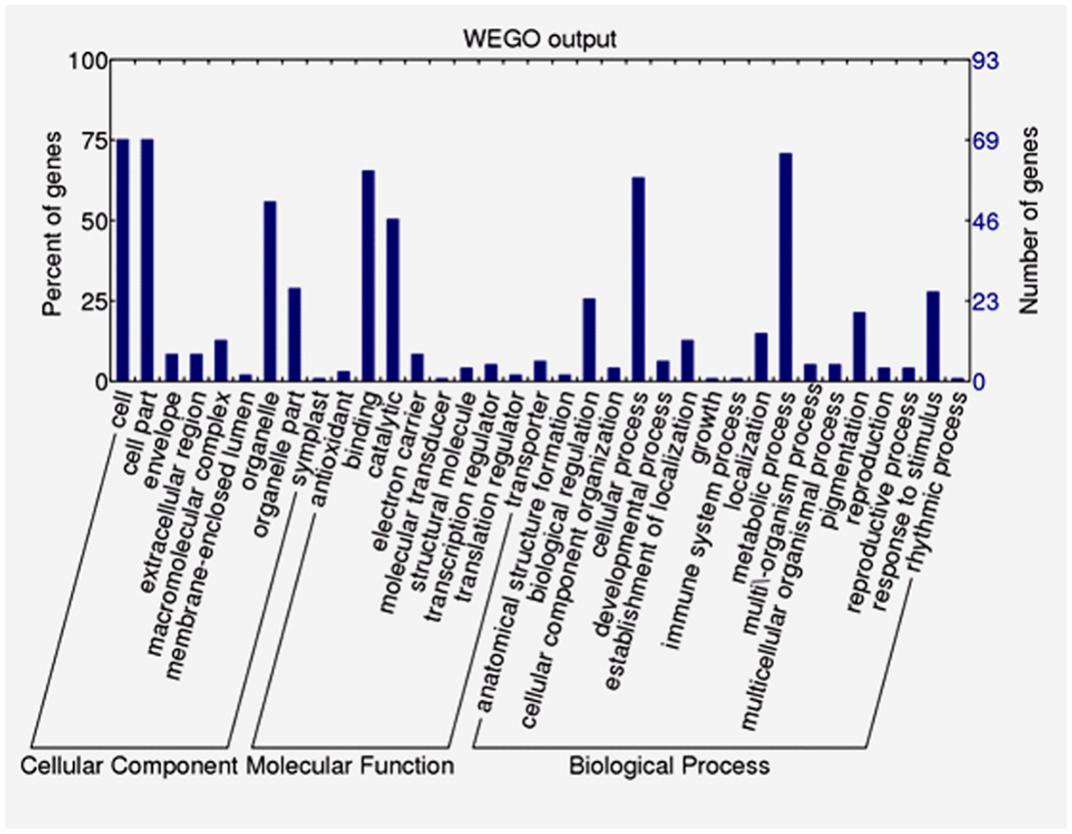
GO function classification of the identified genes expressed specifically or preferentially in the selfed ovules of *Xanthoceras sorbifolium*.

To further evaluate the potential functions of genes in the selfed ovule dataset, Gene Ontology categories were assigned to the 274 specifically or preferentially expressed genes in the selfed ovules. Ninety-three (33.94%) genes were assigned one or more GO terms (S8 Table), and 92 of these were assigned to biological process and were further classified into 17 subcategories (Fig 9). Among the 17 subcategories, metabolic and cellular processes; biological regulation; and response to stimulus were predominant. Under cellular component, cell parts; cell; organelle and organelle part were the largest subcategories. Binding (nucleotide binding, protein binding, chromatin binding) and catalysis were the most abundant subcategories within molecular function (Fig 9).

**Fig 10:**
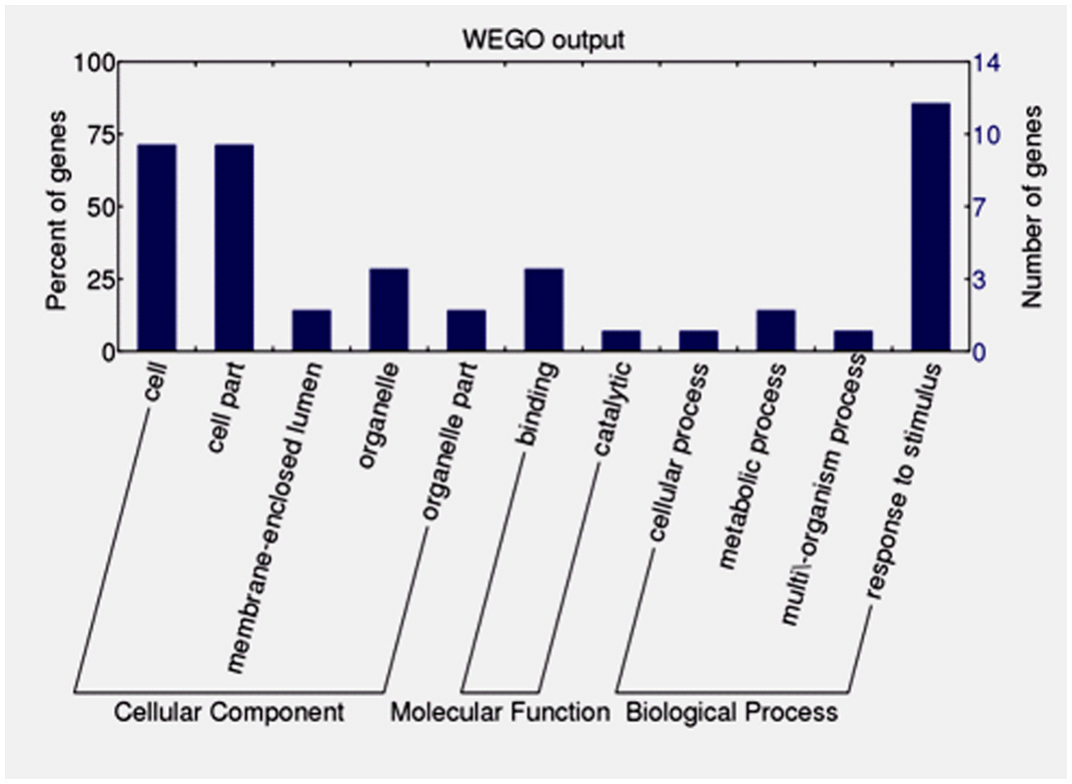
GO function classification of the identified genes expressed specifically or preferentially in the crossed ovules of *Xanthoceras sorbifolium*.

Fourteen unigenes expressed specifically or preferentially in the crossed ovules were assigned with GO terms. In cell component category, cell and cell part subcategories are dominant, followed by organelle, macromolecular complex, and membrane-enclosed lumen. Biological process category includes response to stimulus, metabolic process, cellular process, and multi-organism process. Binding is the largest subcategory within molecular function category (Fig 10).

For large groups of genes, statistically enriched GO terms can give insights into the biological pathways that are likely to be highly active by comparing them to the frequency at which those GO terms appear in the whole transcriptome. A singular enrichment analysis (SEA) [30] was performed to identify the significantly enriched GO terms in genes specifically or preferentially expressed in the selfed ovules of *X*. *sorbifolium*. The results showed that 18 GO terms were overrepresented in the selfed ovules based on the P-value <0.001 and the FDR ≤0.05 cutoffs, which included 15 cellular component categories and 3 molecular function categories (Table 1). The genes involved in the plastid, chloroplast part, plastid part and thylakoid were overrepresented based on the GO cellular component analysis. In the molecular function category, the selfed ovule was enriched in GO terms related to iron ion binding, tetrapyrrole binding and oxidoreductase activity.

**Table 1:** The overrepresented functional GO terms of the genes specifically or preferentially in the selfed ovules of *Xanthoceras sorbifolium*.

**GO term**	**Ontogeny**	**Description**	**Query item number**	**Query total**	**Number in BG/Ref**	**Bg total**	**p-value**	**FDR**
**GO:0046906**	F	tetrapyrrole binding	11	93	143	6809	8.20E-06	0.0012
**GO:0005506**	F	iron ion binding	12	93	186	6809	2.00E-05	0.0015
**GO:0016705**	F	oxidoreductase activity, acting on paired donors, with incorporation or reduction of molecular oxygen	7	93	75	6809	9.80E-05	0.0049
**GO:0009579**	C	thylakoid	12	93	185	6809	1.90E-05	0.0019
**GO:0009522**	C	photosystem I	5	93	23	6809	1.50E-05	0.0019
**GO:0044435**	C	plastid part	19	93	451	6809	5.10E-05	0.0026
**GO:0044434**	C	chloroplast part	19	93	443	6809	4.10E-05	0.0026
**GO:0031976**	C	plastid thylakoid	10	93	167	6809	0.00016	0.0055
**GO:0009534**	C	chloroplast thylakoid	10	93	167	6809	0.00016	0.0055
**GO:0031984**	C	organelle subcompartment	10	93	186	6809	0.00038	0.0097
**GO:0009521**	C	photosystem	5	93	44	6809	0.00038	0.0097
**GO:0009507**	C	chloroplast	27	93	935	6809	0.00089	0.017
**GO:0055035**	C	plastid thylakoid membrane	8	93	140	6809	0.00093	0.017
**GO:0042651**	C	thylakoid membrane	8	93	142	6809	0.001	0.017
**GO:0009535**	C	chloroplast thylakoid membrane	8	93	140	6809	0.00093	0.017
**GO:0009536**	C	plastid	27	93	949	6809	0.0011	0.017
**GO:0034357**	C	photosynthetic membrane	8	93	147	6809	0.0013	0.018
**GO:0044436**	C	thylakoid part	8	93	157	6809	0.0019	0.02

Of 274 unigenes in the selfed ovule dataset, 37 were annotated with a K number to BRITE functional hierarchies, and 26 of 37 unigenes were assigned an EC number (Table 2). The BRITE functional mapping categorized the gene models into 22 KEGG pathways. Photosynthesis; photosynthesis-antenna proteins; cysteine and methionine metabolism; phenylalanine metabolism; and ribosome were the most abundant pathways.

**Table 2:** The *Xanthoceras sorbifolium* selfed ovule specifically or preferentially expressed genes were annotated to BRITE functional hierarchies.

**Gene-ID**	**Ko-ID**	**Enzymes/Proteins**	**EC Number**	**Pathways**
**comp11446_c0_seq1**	K08914	LHCB3; light-harvesting complex II chlorophyll a/b binding protein 3		00196 Photosynthesis—antenna proteins
**comp12163_c0_seq1**	K02638	petE; plastocyanin		00195 Photosynthesis
**comp12271_c0_seq1**	K02975	RP-S25e, RPS25; small subunit ribosomal protein S25e		03010 Ribosome
**comp15623_c0_seq1**	K14498	SNRK2; serine/threonine-protein kinase SRK2	[EC:2.7.11.1]	04075 Plant hormone signal transduction
**comp15703_c0_seq1**	K02639	petF; ferredoxin		00195 Photosynthesis
**comp15750_c0_seq1**	K00430	E1.11.1.7; peroxidase	[EC:1.11.1.7]	00360 Phenylalanine metabolism
**comp16174_c0_seq3**	K15398	CYP86A4S; cytochrome P450, family 86, subfamily A, polypeptide 2/4/7/8 (fatty acid omega-hydroxylase)	[EC:1.14.-.-]	00073 Cutin, suberine and wax biosynthesis
**comp16267_c0_seq2**	K00878	thiM; hydroxyethylthiazole kinase	[EC:2.7.1.50]	00730 Thiamine metabolism
**comp16909_c0_seq1**	K04125	E1.14.11.13; gibberellin 2-oxidase	[EC:1.14.11.13]	00904 Diterpenoid biosynthesis
**comp18656_c0_seq1**	K15813	LUP4; beta-amyrin synthase	[EC:5.4.99.39]	00909 Sesquiterpenoid and triterpenoid biosynthesis
**comp18849_c0_seq4**	K13356	FAR; fatty acyl-CoA reductase	[EC:1.2.1.-]	00073 Cutin, suberine and wax biosynthesis
**comp19771_c0_seq1**	K02152	ATPeVG, ATP6G1; V-type H+-transporting ATPase subunit G	[EC:3.6.3.14]	00190 Oxidative phosphorylation
**comp21740_c0_seq1**	K10111	malK, mtlK, thuK; multiple sugar transport system ATP-binding protein	[EC:3.6.3.-]	02010 ABC transporters
**comp22480_c0_seq1**	K13789	GGPS; geranylgeranyl diphosphate synthase, type II	[EC:2.5.1.1 2.5.1.10 2.5.1.29]	00900 Terpenoid backbone biosynthesis
**comp23147_c0_seq1**	K15746	crtZ; beta-carotene 3-hydroxylase	[EC:1.14.13.129]	00906 Carotenoid biosynthesis
**comp23211_c0_seq1**	K05907	APR; adenylyl-sulfate reductase (glutathione)	[EC:1.8.4.9]	00920 Sulfur metabolism
**comp23344_c0_seq1**	K14498	SNRK2; serine/threonine-protein kinase SRK2	[EC:2.7.11.1]	04075 Plant hormone signal transduction
**comp24050_c0_seq1**	K00454	LOX2S; lipoxygenase	[EC:1.13.11.12]	00591 Linoleic acid metabolism
**comp24115_c0_seq1**	K12153	CYP79A2; cytochrome P450, family 79, subfamily A, polypeptide 2 (phenylalanine N-monooxygenase)	[EC:1.14.13.124]	00460 Cyanoamino acid metabolism
**comp24691_c0_seq1**	K00522	FTH1; ferritin heavy chain	[EC:1.16.3.1]	00860 Porphyrin and chlorophyll metabolism
**comp24825_c0_seq1**	K08905	psaG; photosystem I subunit V		00195 Photosynthesis
**comp24912_c0_seq3**	K05279	E2.1.1.76; flavonol 3-O-methyltransferase	[EC:2.1.1.76]	00944 Flavone and flavonol biosynthesis
**comp26604_c0_seq1**	K02900	RP-L27Ae, RPL27A; large subunit ribosomal protein L27Ae		03010 Ribosome
**comp31612_c0_seq1**	K13034	ATCYSC1; L-3-cyanoalanine synthase/ cysteine synthase	[EC:2.5.1.47 4.4.1.9]	00270 Cysteine and methionine metabolism
**comp32523_c0_seq1**	K00588	E2.1.1.104; caffeoyl-CoA O-methyltransferase	[EC:2.1.1.104]	00360 Phenylalanine metabolism
**comp32688_c0_seq1**	K00547	mmuM; homocysteine S-methyltransferase	[EC:2.1.1.10]	00270 Cysteine and methionine metabolism
**comp33345_c0_seq1**	K00430	E1.11.1.7; peroxidase	[EC:1.11.1.7]	00360 Phenylalanine metabolism
**comp5793_c0_seq1**	K15086	TPS14; (3S)-linalool synthase	[EC:4.2.3.25]	00902 Monoterpenoid biosynthesis
**comp6957_c0_seq1**	K03236	EIF1A; translation initiation factor 1A		03013 RNA transport
**comp7253_c0_seq1**	K02692	psaD; photosystem I subunit II		00195 Photosynthesis
**comp7608_c0_seq1**	K02905	RP-L29e, RPL29; large subunit ribosomal protein L29e		03010 Ribosome
**comp7631_c0_seq1**	K01761	E4.4.1.11; methionine-gamma-lyase	[EC:4.4.1.11]	00270 Cysteine and methionine metabolism
**comp7882_c0_seq1**	K08909	LHCA3; light-harvesting complex I chlorophyll a/b binding protein 3		00196 Photosynthesis—antenna proteins
**comp8062_c0_seq1**	K00090	E1.1.1.215; gluconate 2-dehydrogenase	[EC:1.1.1.215]	00030 Pentose phosphate pathway
**comp8280_c0_seq1**	K02126	ATPeF0A, MTATP6; F-type H+-transporting ATPase subunit a	[EC:3.6.3.14]	00190 Oxidative phosphorylation
**comp8351_c0_seq1**	K01188	E3.2.1.21; beta-glucosidase	[EC:3.2.1.21]	00460 Cyanoamino acid metabolism
**comp9794_c0_seq2**	K08912	LHCB1; light-harvesting complex II chlorophyll a/b binding protein 1		00196 Photosynthesis—antenna proteins

### Transcription factors in the fertilized ovules of *X*. *sorbifolium*

**Fig 11:**
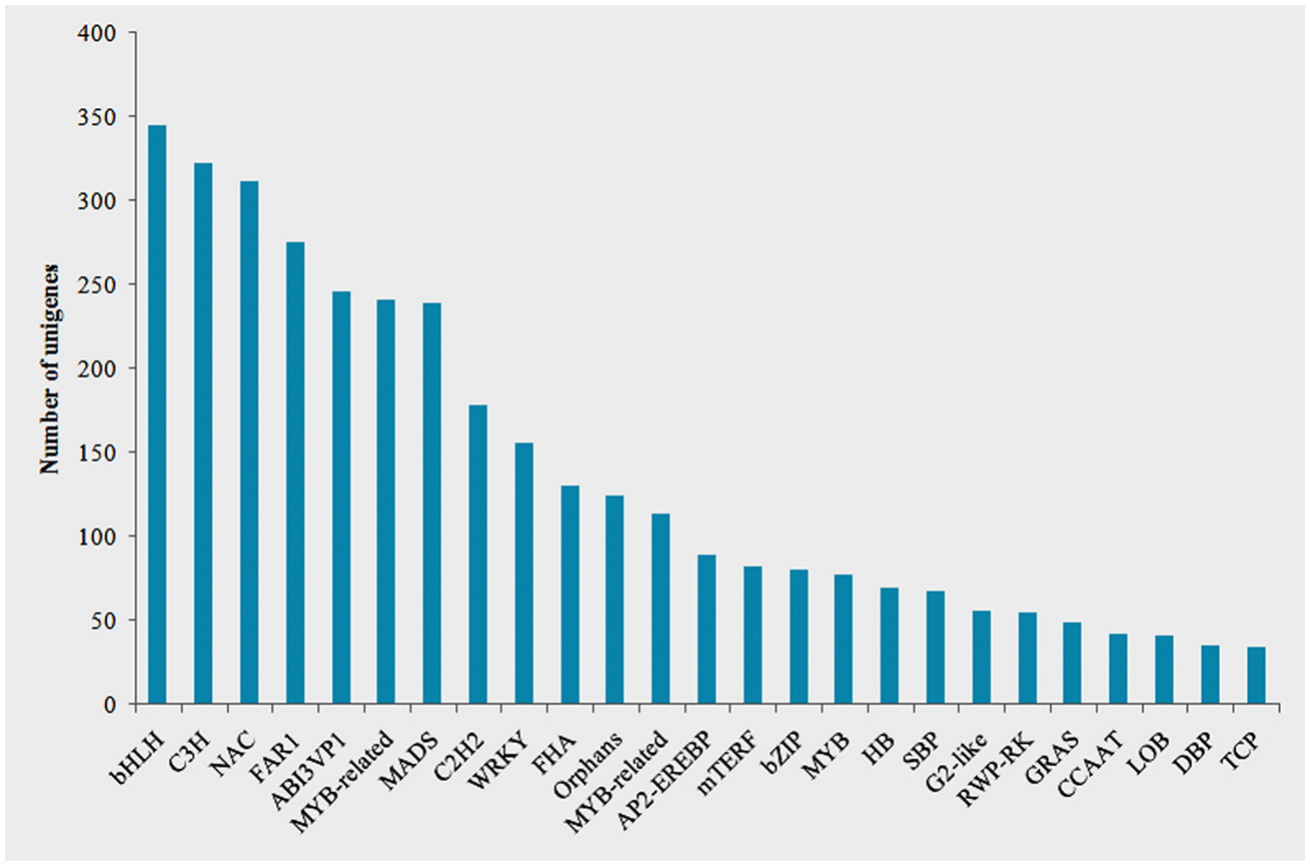
Top 25 transcription factor families of the *Xanthoceras sorbifolium* selfed ovule dataset. The X-axis indicates the top 25 TF families. The Y-axis indicates the number of unigenes assigned to a specific TF family.

Transcription factors (TFs) play a pivotal role in regulating the spatial and temporal expression of genes in all living organisms. This regulation ensures accurate development and functioning of an organism. To understand transcription factor expression patterns in the fertilized ovules of *X*. *sorbifolium*, all the assembled transcripts were aligned with known TF protein sequences of other sequenced plants listed in PlnTFDB (E-value ≤ 1e-10) using BLASTX. In total, 3,812 putative TF-encoding transcripts, distributed over at least 60 families, were identified, representing 10.55% of the total ovule transcripts detected in the present study. The top 25 TF gene families are depicted in Fig 11. The largest TF family was FAR1, which contained 509 unigenes. The next largest families were PHD, MADS, C3H, bHLH, MYB, NAC, and WRKY family TFs.

TF identification is useful for studying the transcriptional regulatory switches involved in plant morphology and functional competence and also in generating responses to changing condition. Hence, it was of great interest to perform a comparative analysis of TF gene expression between the selfed and crossed ovules in an LSI species. Of 274 unigenes expressed specifically or preferentially in the selfed ovules of *X*. *sorbifolium*, 28 encode for putative transcription factors belonging to 12 different families (Table 3). The most frequently represented genes in the largest category encode for the FAR1 TF family, which contains eight genes. The most highly expressed TF-encoding transcripts with RPKM values of above 10 in the selfed ovules encode the FAR1, HB, NAC, and MYB families. Among 42 genes expressed specifically in the crossedovules, 4 encode for NAC, FAR1, MYB and LIM TF families.

**Table 3:** Twenty-eight specifically or preferentially expressed unigenes in the selfed ovules of *Xanthoceras sorbifolium* encode for the putative transcription factors belonging to 12 different families. RPKM: Reads per Kilobase per Million reads.

**Gene_ID**	**Transcription factor families**	**RPKM**
comp7866_c0_seq1	FAR1	70.68928969
comp13650_c0_seq1	HB	37.96544751
comp13995_c0_seq1	FAR1	16.56510458
comp10460_c0_seq2	FAR1	16.21917041
comp7496_c0_seq1	FAR1	14.15901078
comp10091_c0_seq2	NAC	13.74973919
comp15623_c0_seq1	HB	13.23787232
comp15750_c0_seq1	MYB	11.74753425
comp16204_c0_seq1	bZIP	8.663881644
comp12230_c0_seq1	C3H	8.615953788
comp12503_c0_seq1	OFP	8.326351607
comp6837_c0_seq1	FAR1	7.182740113
comp16650_c0_seq1	C2H2	6.861621657
comp17938_c0_seq1	C2C2-CO-like	6.756090309
comp8243_c0_seq1	FAR1	5.846955337
comp17767_c0_seq2	FHA	5.838119077
comp13002_c0_seq2	HB	5.48346443
comp17703_c0_seq2	SBP	5.327418492
comp12970_c0_seq1	HB	5.121261816
comp12417_c0_seq1	MYB-related	4.838350968
comp8062_c0_seq1	HB	4.269904026
comp8351_c0_seq1	HB	4.187594785
comp7986_c0_seq2	MYB-related	4.152074582
comp18100_c0_seq2	PLATZ	3.765540213
comp17167_c0_seq1	C2H2	3.757697173
comp17403_c0_seq2	MYB-related	3.163581425
comp5928_c0_seq1	FAR1	2.7641009
comp6110_c0_seq2	FAR1	2.134615466

## Discussion

Historically, most studies on SI have focused on the dynamics of interactions between pollen and the stigma or the style, with little attention given to events occurring in the ovary. Late-acting SI has been neglected and was often treated as an anomaly of limited importance by researchers studying the conventional SI systems, in which SI barriers occur at stigmatic and stylar levels of the pistil. This situation has recently changed because OSI was revealed in many angiosperm species [20,24,27,35–44]and hence is known to play an important role, as do other forms of SI, in reducing inbreeding and its harmful effects [45]. However, in most instances the critical structural and histological investigations of ovule development were required to distinguish whether self-sterility results from a true OSI based on self-recognition with major gene control or due to the effects of early acting inbreeding depression (EID), caused by the expression of deleterious recessive alleles. Discrimination between LSI and EID may be a difficult task [21]. This situation is particularly contentious for the cases of post-zygotic rejection of selfed pistils in presumed LSI species, as in *X*. *sorbifolium*.

To distinguish between late-acting SI and early acting ID, one of the principal criteria is the timing of ovule abortion [21]. Early acting ID is expected to cause embryo failure at a variety of developmental stages, whereas a uniform failure of ovules at a single developmental stage would be interpreted as late-acting SI. The morphological investigations in the present study sufficiently demonstrate the occurrence of double fertilization in all the selfed ovules of *X*. *sorbifolium* and the uniform failure of zygotes arising from self-pollination at an initial stage prior to cell division, with the early stages of free-nuclear endosperm formation apparently proceeding normally. Similar post-penetration events in selfed ovules were also observed in other LSI species studied, such as in Bignoniaceae species [37–38,46], *Pseudowintera axillaris* [20], *Gasteria verrucosa* [47], *Narcissus triandrus* [48], and *Ipomopsis aggregate* [41]. No embryo divisions occurred in any of these species and these plants show the phenomenon of ‘resting zygotes’. The failure of zygotic division is followed by the rejection of an entire ovule.

The morphological observations in *X*. *sorbifolium* also indicated that self-pollen or pollen tubes elicit a reduction in embryo sac size, quick deposition of thick, lignified cell wall, and pronounced accumulation of tannin-like substances in the outer integument in comparison to cross-pollen or pollen tubes. These results suggested that the process of self-recognition and rejection in *X*. *sorbifolium* may entail long-distance signaling between pollen or pollen tubes and ovarian tissues, which results in the modification of post-pollination stimulatory functions of pollen or pollen tubes on ovule development. Late-acting SI may start early, and self-pollen tubes growing in the style may project ‘hostile’ or ‘adverse’ signals that may set in motion a subsequent chain of events that lead to ovule rejection.

Long-distance signaling from the pollen tube to the ovules has been implicated in some LSI taxa in which the presence of incompatible pollen or pollen tubes influence ovule integument development, embryo sac viability, starch metabolism, and transmitting tissue secretion in ovarian tissue before and after penetration by pollen tubes [41,47]. It has been suggested for several species exhibiting LSI that self-pollen tubes do not provide the appropriate signals for stimulation of ovule and seed development. In *Gasteria verrucosa* [47], *Theobroma cacao* [22], and *Asclepias exaltata* [26], integumentary growth fails to proceed normally after entry of a self-pollen tube into the ovule. Sears suggested that interaction of compatible pollen tubes with integuments might be important for stimulation of normal seed development [47]. In *Prunus dulcis*, embryo sac development was strongly affected by pollen tube activity in the pistil. Cross-pollen tubes had a greater stimulatory effect than self-pollen tubes and irregularities in embryo sac development were more frequent after self-pollination [49]. Sage et al. showed that embryo sac degeneration after self-pollination might result from the absence of a required stimulus for normal ovule development in *Narcissus triandru* [48]. Sage et al. reported that ovules in self-pollinated flowers of *Ipomopsis aggregate* indicated an absence of embryo sac expansion, little starch storage, disorderly development of the integumentary tapetum and adjacent cells before pollen tube entry into the ovary [41]. Indoleacetic acid, gibberellic acid, ethylene, and ethylene precursors have been posited to play a role in post-pollination stimulation events by pollen tubes [50].

Where free-nuclear endosperm and the resting zygote develop, the ovule must possess or produce some substance that actively inhibits the growth of selfed endosperm and division of the zygotes in the LSI species. Early priming for ovule degeneration triggered by possible adverse signals from self-pollen tubes might be revealed by searching for early molecular indicators of biological processes. The present study identified 274 genes predicted to be specifically or preferentially expressed in the selfed ovules of *X*. *sorbifolium* using high-throughput next-generation sequencing technology to perform an RNA-seq analysis. It is likely that at least some of these genes function in pollen-ovule interactions and LSI mechanisms. This study represents the first genome-wide identification of genes expressed in the ovules of a late-acting SI species.

The genes expressed specifically or preferentially in the selfed ovules at 5 DAP were predicted to encode proteins that may perform crucial functions in the switch from normal to aberrant development of ovules. Our analysis found that overrepresented functional categories in the transcripts expressed specifically or preferentially in selfed ovules include signal transduction mechanisms, secondary metabolites biosynthesis, transcription, inorganic ion transport and metabolism. We hypothesize that at least some of these genes are potentially involved in the pollination compatibility responses and the LSI process.

### Cell-cell communication and signal transduction possibly implicated in LSI responses

Genes predicted to function in cell-cell communication and signal transduction are of particular interest in the context of pollen-ovule interactions and LSI. The selfed ovule dataset contained a significant proportion of genes in these categories. The most noteworthy are genes encoding Ca^2+^/calmodulin-dependent protein kinase, serine/threonine protein kinase, serine/threonine protein phosphatase.

Protein phosphorylation/dephosphorylation by specific protein kinases/phosphatases is one of the most important mechanisms whereby cells respond to extracellular signals [51]. It is well known that protein phosphorylation events play a crucial role in the signaling cascade of *Papave*r GSI and *Brassica* SSI. Interaction between pollen S-ligand (SCR/SP11) and its cognate receptor (SRK) results in transphosphorylation of the kinase domain of SRK, leading to activation of elements of a signaling cascade in *Brassica* SI [52]. Phosphorylation of the p26 pyrophosphatases and p56 (a mitogen-activated protein kinase, MAPK) is crucial for the SI response in *Papaver* [53–55]. MAPKs are Ser/Thr protein kinases that are activated by phosphorylation. Activated MAPKs trigger diverse signaling cascades in response to a variety of signals and stimuli [56]. In *Nicotiana alata*, a species with S-RNase SI, a pollen Ca^2+^-dependent protein kinase has been shown to specifically phosphorylate the S-RNase [57]. It is particularly notable that three unigenes encoding protein kinase and three encoding protein phosphatase were observed in the *X*. *sorbifolium* selfed ovule dataset. Among these six potential candidate genes encoding signaling-related components, 3 (comp13650_c0_seq1, comp15623_c0_seq1 and comp17403_c0_seq2) were confirmed using PCR in the selfed and crossed ovules. Although we are far from understanding the precise role of these protein kinases and phosphatases in the *X*. *sorbifolium* LSI mechanisms, it is likely that these proteins are potentially involved in the LSI process and that their roles may be analogous to those in the incompatibility responses of the other species studied.

Ca^2+^ ions are a most versatile second messenger used in signal transduction in all eukaryotic organisms. In plants, temporally and spatially distinct changes in cytosolic Ca^2+^ concentrations that are evoked in response to different stimuli, designated as “Ca^2+^ signatures”, represent a central mechanistic principle to present defined stimulus-specific information [58]. The Ca^2+^ signatures are detected, decoded and transmitted downstream by Ca^2+^ sensors [59–60]. Several classes of calcium-sensing proteins have been identified in higher plants, including calmodulin, calmodulin-like, calcineurin B-like proteins, and calcium-dependent protein kinases [61–62]. The *X*. *sorbifolium* selfed ovule dataset contains a unigene predicted to encode Ca^2+^/calmodulin-dependent protein kinase. This gene implicates the involvement of Ca^2+^-mediated signaling and Ca^2+^-dependent protein kinase in the *X*. *sorbifolium* LSI response.

### Transcription factors likely involved in coordinated activation of genes related to LSI

The processes underlying the pollen-ovule interaction and LSI require the concerted action of genes that are regulated by transcription factors. Transcription factors are sequence-specific DNA-binding proteins that may simultaneously function as an activator of one set of functionally related genes and a repressor of others. TFs are responsible for selective gene regulation and are often expressed in a tissue-specific, developmental stage-specific or stimulus-dependent manner [63].

We identified 28 TF genes in the selfed ovule dataset that are likely to play critical regulatory roles in controlling developmental events unique to the selfed ovule development of *X*. *sorbifolium*. Therefore 28 is the lower limit of the number of regulators in the selfed ovule dataset because of the stringent filtering process we used in analyzing RNA-seq datasets generated in this study. Most of these TFs (58%) belonged to three families: FAR1 (FAR-RED IMPAIRED RESPONSE1), HB and MYB. Among these families, the FAR1 genes (8) form the largest category. FAR1 functions as a positive and key transcription factor in directly regulating chlorophyll biosynthesis in Arabidopsis. FAR1 and FHY3 (FAR-RED ELONGATED HYPOCOTYL 3) work together to modulate phytochrome A (phyA) nuclear accumulation and phyA responses through directly activating gene expression of a pair of downstream targets, *FHY1* and *FHY1-LIKE*. FHY1 and FHL are two small plant-specific proteins required for nuclear accumulation of light-activated phyA. We observed 3 unigenes predicted to encode chlorophyll a-b binding protein in the selfed ovules dataset. This suggests a general trend to enhance photosynthesis in the selfed ovules of *X*. *sorbifolium* during incompatible pollen-ovule interactions.

Most MYB proteins function as transcription factors with varying numbers of MYB domain repeats conferring their ability to bind DNA [64–65]. MYB proteins can be divided into four classes. Most plant MYB genes encode proteins of the R2R3-MYB class [64]. Numerous R2R3-MYB proteins are involved in the control of plant-specific processes including: primary and secondary metabolism, cell fate and identity, developmental processes, and responses to biotic and abiotic stresses [65]. For instance, the R2R3-MYB proteins encoded by *AtMYB5* and *AtMYB23* regulate tannin biosynthesis in Arabidopsis [66]. AtMYB5 also regulates outer seed coat differentiation. AtMYB52, AtMYB54 and AtMYB69 are proposed to regulate lignin, xylan and cellulose biosynthesis [67]. AtMYB58, AtMYB63 and AtMYB85 activate lignin biosynthesis in fibers and/or vessels [68], whereas AtMYB68 negatively regulates lignin deposition in roots [69]. Our histological analyses indicated that cell wall lignification and accumulation of tannin-like substances in the outer integument occurred more quickly and more heavily after self-pollination than cross- pollination in *X*. *sorbifolium*. We hypothesize that some of the MYB genes detected by the present study are potentially involved in tannin and lignin biosynthesis in the integuments of *X*. *sorbifolium*.

Phosphorylation is important in determining MYB protein activity. The transcriptional activity of the R2R3-MYB PtMYB4 protein in *Pinus taeda* is positively regulated by PtMAPK6, which phosphorylates a Ser in the C-terminal activation domain, and similar phosphorylation might regulate other R2R3-MYB proteins, such as AtMYB46 in Arabidopsis [70]. It is likely that some specifically or preferentially expressed protein kinases in the selfed ovules in *X*. *sorbifolium* are also involved with regulation of MYB protein activity.

The selfed ovule dataset also contains many TF genes not previously identified as ovule-specific in other species. These include OFP, SBP, SNF2 and PLATZ. The identification of novel TF genes in the *X*. *sorbifolium* selfed ovule dataset is particularly interesting as this species possesses an LSI system, which most likely operates through a different mechanism from other conventional SI and self-compatibility (SC) systems. Therefore, the *X*. *sorbifolium* selfed ovule dataset is expected to contain TF genes potentially involved in mediating the female side of LSI.

### Biosynthesis of secondary metabolites possibly related to rejection of selfed ovules

Incompatible pollen-pistil interactions are sometimes associated with the accumulation of intermediates of secondary metabolite pathways [71], such as the phenylpropanoid pathway, which also occurs in the plant’s response to pathogens and stress [72]. The *X*. *sorbifoilum* selfed ovule dataset contains a relatively large number of cytochrome P450 unigenes. P450 enzymes are an ancient superfamily of heme-containing monooxygenase proteins found in all domains of life [73], most of which catalyze NADPH and O_2_-dependent hydroxylation reactions. Plant P450s are involved in a wide range of biochemical pathways, including those devoted to the synthesis of the following: lignin intermediates; phenylpropanoids; alkaloids; terpenoids; lipids; cyanogenic glycosides; glucosinolates; and plant growth regulators such as gibberellins, jasmonic acid, and brassinosteroids [72,74 **–** 75]. The CYP superfamily has a total of 977 families, of which 69 are present in animals [76]. The CYP2 gene family is the largest and most complex of the 18 CYP gene families in vertebrates. The number of genes per CYP2 subfamily is variable and can be quite large in some species. CYP2s play a significant role in the metabolism of a variety of exogenous and endogenous compounds [77–78]. The present studies identified 4 unigenes homologous to members of CYP2 family. It will be of interest to investigate whether these CYP2 genes are involved in pollen-ovule interactions and LSI.

## Conclusion

The present study demonstrated that there were no significant differences in structural and histological features between the selfed and crossed ovules during the earliest stages of development. After 5 DAP, some of the selfed ovules ceased developing and showed visible signs of degeneration. These observations suggest that 5 DAP is likely a turning point for development of selfed ovules and that the reprogrammed gene expression at this stage possibly represents the molecular mechanisms essential for the rejection of the selfed ovules. The comparative *de novo* transcriptome analysis of the selfed and crossed ovules at 5 DAP using high-throughput next-generation sequencing technology resulted in the identification and functional classification of 274 genes that were specifically or preferentially expressed in the selfed ovules. Although the biological roles of these genes have yet to be determined, it is likely that at least some of the genes expressed specifically or preferentially in the selfed ovule have functions related to the development of the selfed ovules and LSI mechanisms. To our knowledge, there were no data on genes related to the LSI process prior to our study. This study represents the first genome-wide identification of genes expressed in the fertilized ovules of a late-acting SI species. The valuable genomic resources obtained here will trigger new interesting research on molecular mechanisms of LSI and promote a better understanding of LSI more comparable to those of conventional GSI and SSI systems.

## Supporting Information

S1 Table 274 genes that were specifically or preferentially expressed in the selfed ovules of *Xanthoceras sorbifolium*. (XLSX)

S2 Table 42 genes that were specifically or preferentially expressed in the crossed ovules of *Xanthoceras sorbifolium*. (XLSX)

S3 Table A list of real- time PCR primers used in the present study. (XLSX)

S4 Table GO terms assigned to 6,809 unigenes. (XLSX)

S5 Table Unigenes assigned with KO number and EC number. (XLSX)

S6 Table The BRITE functional mapping categorized the unigenes into 252 KEGG pathways. (XLSX)

S7 Table Seventy-six unigenes expressed specifically or preferentially in the selfed ovules were assigned with KOG annotation and classification. (XLSX)

S8 Table GO terms were assigned to the genes expressed specifically or preferentially in the selfed ovules of *Xanthoceras sorbifolium*. (XLSX)
